# PIPE-T: a new Galaxy tool for the analysis of RT-qPCR expression data

**DOI:** 10.1038/s41598-019-53155-9

**Published:** 2019-11-26

**Authors:** Nicolò Zanardi, Martina Morini, Marco Antonio Tangaro, Federico Zambelli, Maria Carla Bosco, Luigi Varesio, Alessandra Eva, Davide Cangelosi

**Affiliations:** 1 Laboratory of Molecular Biology, IRCCS Istituto Giannina Gaslini, Via Gerolamo Gaslini 5, 16147 Genova, Italy; 2 Institute of Biomembranes, Bioenergetics and Molecular Biotechnologies, National Research Council, Via Giovanni Amendola, 122/O, 70126 Bari, Italy; 3 Department of Bioscience, University of Milan, Via Celoria 26, 20133 Milano, Italy

**Keywords:** Software, Statistical methods, Transcriptomics

## Abstract

Reverse transcription quantitative real-time polymerase chain reaction (RT-qPCR) is an accurate and fast method to measure gene expression. Reproducibility of the analyses is the main limitation of RT-qPCR experiments. Galaxy is an open, web-based, genomic workbench for a reproducible, transparent, and accessible science. Our aim was developing a new Galaxy tool for the analysis of RT-qPCR expression data. Our tool was developed using Galaxy workbench version 19.01 and functions implemented in several R packages. We developed PIPE-T, a new Galaxy tool implementing a workflow, which offers several options for parsing, filtering, normalizing, imputing, and analyzing RT-qPCR data. PIPE-T requires two input files and returns seven output files. We tested the ability of PIPE-T to analyze RT-qPCR data on two example datasets available in the gene expression omnibus repository. In both cases, our tool successfully completed execution returning expected results. PIPE-T can be easily installed from the Galaxy main tool shed or from Docker. Source code, step-by-step instructions, and example files are available on GitHub to assist new users to install, execute, and test PIPE-T. PIPE-T is a new tool suitable for the reproducible, transparent, and accessible analysis of RT-qPCR expression data.

## Introduction

Quantitative real-time polymerase chain reaction (qPCR) is a routinely used technique for the detection of specific nucleic acids, RNA expression profiling, quantification of DNA and DNA methylation, and validation of microarray hybridization data^1^. Reverse transcription qPCR (RT-qPCR) is an accurate, sensitive, and fast method to quantify gene expression from qPCR experiments^2^, and is widely accepted as the Golden Standard for the analysis of gene expression^1,3^. Briefly, RT-qPCR measures the expression of a set of target RNAs through repeated cycles of sequence-specific amplification followed by expression measurements^4^. The cycle at which the observed expression first exceeds a user-specified threshold is commonly called the threshold cycle (Ct) or quantification cycle. The Ct values of the target RNAs represent a quantitative assessment of gene expression and are often treated as the raw data for subsequent analyses^4^. Two methods can be used to quantify gene expression from the Ct value: the absolute and the relative quantification^3^. In the absolute quantification, a standard curve is used as reference calibrator. In the relative quantification, the signal is related to the expression of a user-specified group^3^. Therefore, the difference between the two approaches depends on the data used as reference calibrator to which relating the signal.

In many RT-qPCR experiments not all Ct values can be numerically defined. For example, when the starting RNA abundance is too low, or an off-target product is amplified, or no reliable Ct can be determined, the corresponding Ct value cannot be quantified numerically and is flagged as missing value^5^. Handling missing data is a crucial step in the analysis of RT-qPCR experiments because procedures used in the subsequent analyses of these data are based on statistics that are unable to handle both numeric and missing values^4^. Imputation is an established technique to solve the problem^6^. Imputation substitutes a missing value with a rationally selected numeric value^4^. K-nearest neighbors (KNN)^6^, maximum Ct plus one cycle (Mestdagh)^7^, and cubic spline interpolation (Cubic)^1^ are known methods to impute missing values in RT-qPCR data^5,6^.

Another key step in the analysis of RT-qPCR data is the assessment of true biological changes associated with the phenomenon or disease of interest. In fact, biological changes are often masked by nonspecific technical variability introduced in the data during the experimental procedure^6^. Data normalization is expected to reduce/eliminate any technical variability without affecting the true biological results^6^. Global mean^8^, DeltaCt based on universal normalizers^9^, Modified global mean^10^, Quantile^9^, and Rank Invariant^9^ are among the most accepted methods used for RT-qPCR data normalization^5^.

RT-qPCR experiments allow measuring the expression of several transcripts in parallel using high-density plates^9^. Plates have been used in several explorative studies to find novel biomarkers from the analysis of different diseases, tissues, experimental conditions, and cell types^3,5,6^. The large number of studies published in the literature stimulated companies to develop commercial technologies to perform RT-qPCR experiments^3^. For each experiment, these technologies generate textual reports summarizing a number of experimental parameters and data such as feature name, quality control flags, and Ct values. Different technologies generate reports that can be of different format. According to our experience, SDS, EDS, and OpenArray are among the most used file formats for reporting results of RT-qPCR experiments.

Although the computational procedures and technologies for analyzing RT-qPCR data are well established, the heterogeneity of the assays employed in RT-qPCR experiments and the lack of a consensus on the best normalization system and on the missing values imputation approach to adopt makes it hard to set up a standardized analysis procedure^6^. Furthermore producing high quality publications and reproducible data are among the most critical pitfalls of qPCR experiments^11^.

Several open-access software packages, tools, and web applications, such as R packages, have been proposed in the last years for the analysis of RT-qPCR data^1^. HTqPCR is a well-known open source R\Bioconductor package for the high-throughput analysis of RT-qPCR data^9^. It provides several functions and parameter options for assessing the quality of the experiment, filtering unreliable data, normalizing raw data, finding potential candidate biomarkers, and visualizing RT-qPCR data^9^. However, R-based analysis suffers from some known limitations. First of all, analysis procedures are implemented in several packages lacking a unified framework. Second, users with biological background who want to use the functionalities of R packages need non-trivial coding skills. Furthermore, the lack of a simple framework for reusing, sharing, and communicating experimental procedures and results limits reproducibility, transparency, and accessibility of R-based analysis^12^.

Galaxy is an open, collaborative, web-based, genomic workbench for a reproducible, transparent, and accessible science^12^. Galaxy provides a very active developer community. More than 6746 public tools and workflows are freely available in the Galaxy Tool Shed repositories^12^. New tools and workflows are easily deployable in the Galaxy repositories. To this purpose, Galaxy offers fresh installations of R and Python environments, a fast dependency resolver, a step-by-step documentation, a simple graphical interface, and GitHub integration^13^. However, to the best of our knowledge, no Galaxy tool or workflow has been reported to date for analyzing RT-qPCR data.

In the present work, we developed *pipette* (PIPE-T), a new tool for analyzing RT-qPCR expression data integrating the functionalities implemented in various R packages into one unified, reusable, transparent, accessible, and easy to use Galaxy wrapper.

## Methods

### Overview of the main procedures implemented by PIPE-T

PIPE-T implements the relative quantification method using the R language and computing environment^14^.

To start a PIPE-T analysis, users must upload two input files:

- A List collection of tab-separated text files for all samples generated as report of the RT-qPCR experiment (ListOfFile).
- A tab-separated text file associating each filename in ListOfFile with a treatment group (FileTreatment).

**Figure 1:**
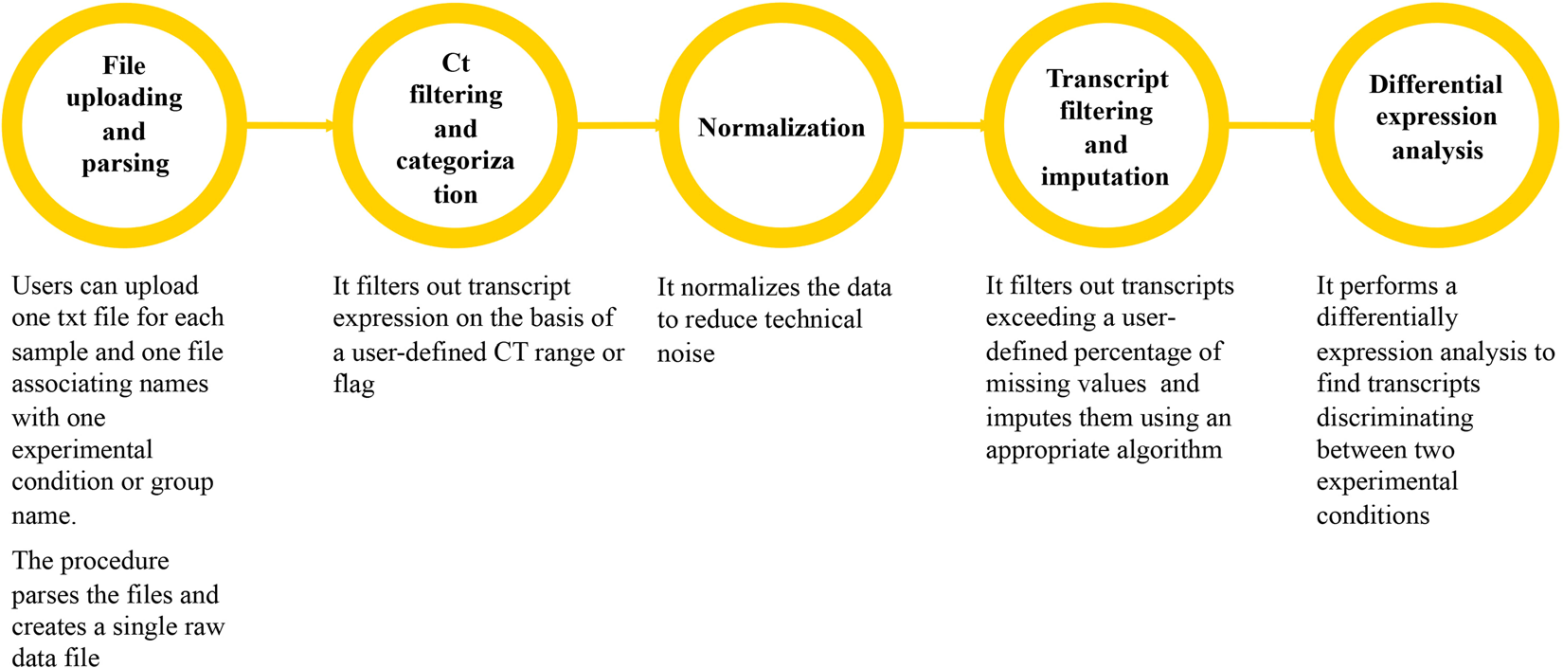
Schematic representation of the analysis procedures implemented in PIPE-T. Input files are uploaded and parsed for initiating the analysis. Transcripts are categorized according to a user-defined range of values and\or quality control flag to label unreliable Ct values. Ct values are normalized to reduce\remove technical variability in the data. Transcripts are filtered out according to a user-specified maximum number of missing values to maintain the bias as low as possible. Imputation is applied to handle missing values. Transcripts discriminating between two treatments are identified for subsequent analyses.

Five distinct computational procedures are implemented in PIPE-T. Procedures are summarized in Fig. 1 and a detailed description of each procedure is provided in the following sections.

The execution of PIPE-T outputs the following output files:

- A tab-separated text file containing the raw Ct values for every sample and transcript
- A PNG file showing the distribution of the Ct values of every samples obtained after the Ct filtering and categorization step visualized as sequence of boxplots.
- A tab-separated text file containing the normalized Ct values
- A PNG file showing the cumulative distribution plot before and after data normalization of the coefficient of variation of every transcript.
- A PNG file showing the distribution of the normalized Ct values visualized as sequence of boxplots.
- A tab-separated text file containing data after imputation
- A tab-separated text file containing the results of the differential expression analysis.

### File uploading and parsing

Heterogeneity of assays quantifying RT-qPCR gene expression is often associated with heterogeneity of the file formats reporting data summarizing the results of the RT-qPCR experiment. Hence, it is crucial that the user uploads files whose content is compliant with the file format parsable by PIPE-T before running any PIPE-T analysis.

“Upload File from your computer” is a Galaxy tool that allows uploading files into Galaxy. This tool is available on any fresh Galaxy instance or on the main Tool Shed repository^15^.

PIPE-T processes tab-separated text files containing a dot as decimal separator uploaded with “Upload File from your computer” tool. The formats supported by PIPE-T are:

- Applied Biosystems Sequence Detection Systems (SDS)
- ThermoFisher Experiment Detection Systems (EDS)
- Applied Biosystems OpenArray (OpenArray)
- Roche LightCycler (LightCycler)
- Bio-Rad CFX (CFX)
- Fluidigm Biomark Table format (BioMark)
- User-formatted plain text (Plain)

SDS, OpenArray, LightCycler, CFX, BioMark, and Plain are HTqPCR R package^9^ parsable file formats. We updated the parsing procedure to adapt it working with R 3.5.0 and tab-separated text files. We extended the list of the parsable file formats including the possibility of processing EDS format, which is one of the most used by Thermo Fisher Scientific real-time qPCR instruments.

FileTreatment should have only two columns named SampleName and Treatment. The column named SampleName lists the name and the extension of the files uploaded into the ListOfFile collection. The column named Treatment associates each sampleName with an experimental condition or group of interest. Group specification is necessary since PIPE-T implements the relative quantification method to analyze data from RT-qPCR experiments. PIPE-T admits the specification of two treatment groups. In the GitHub documentation we provided a checklist of recommendations to help users formatting their input files and checking that these files contain sufficient data to run PIPE-T without errors.

If file format is correct, PIPE-T populates a qPCRset object containing the following data for each transcript and sample:

- Raw Ct\Cq value,
- Value of the internal quality control flag,
- Transcript and sample names,
- FeatureCategory

Data parsing and qPCRset object generation are carried out using the *readCtData* function of the HTqPCR R package^9^.

### Ct filtering and categorization

Feature categorization is a procedure for describing the level of reliability of a transcript and can be used to filter out features whose expression is not sufficiently reliable^9^. HTqPCR package defines three possible categories: “Undertermined”, “Unreliable”, and “OK”^9^. “Undetermined” is used to flag Ct values above a user-defined threshold, and “Unreliable” indicates Ct values that are so low as to be estimated by the user to be problematic^9^.

By default, only Ct values labeled as “undetermined” in the input data files are placed into the “Undetermined” category, and the rest are classified as “OK”^9^.

The FeatureCategory for a transcript can be altered on the basis of two criteria^9^:

- **Range of Ct values**. Some Ct values might be too high or too low to be considered a reliable measure of gene expression in the sample and, therefore, should not be marked as “OK”.
- **Flags**. Depending on the qPCR input, the values might have associated flags, such as “Passed” or “Failed”, which are used for assigning categories.

PIPE-T implements the two criteria allowing users to set up a range of Ct values and a List button. Any Ct value exceeding the user-defined range is categorized as “Unreliable”. Users can force PIPE-T to check internal control flag status. In this case, the FeatureCategory for a transcript is replaced by an “Undetermined” if the transcript did not pass internal quality control.

PIPE-T uses FeatureCategory labels to replace any Ct values corresponding to “Undertermined” and “Unreliable” with a not accessible value (NA).

These operations are carried out using *setCategory* and *filterCategory* functions of HTqPCR package^9^.

### Normalization

Data normalization allows to minimize unwanted systematic technical and experimental variation in the data for better appreciating true biological changes^16^.

PIPE-T offers six different normalization options that are listed below:

- Global mean^8^
- DeltaCt^9^
- Modified global mean^10^
- Quantile^9^
- Norm Rank Invariant^9^
- Scale rank invariant^9^.

Global mean, quantile, norm rank invariant, and scale rank invariant were already implemented in HTqPCR R package^9^. However, as Norm Rank Invariant and Scale rank invariant worked only if missing values were absent, we extended the procedure substituting any missing value with a numeric value using the na.spline function implemented in the zoo R package^17^. D’haene and collegues showed the benefits of using the geometric mean for the normalization of microRNA expression data by introducing the so-called modified global mean method^10^. For these reasons, we integrated the modified global mean method in PIPE-T.

PIPE-T supports the deltaCt method. Housekeeping genes can be specified by the user or can be estimated by the geNorm or NormFinder methods implemented in the NormqPCR R\Bioconductor package^18^. When geNorm is selected, PIPE-T identifies candidate normalizers taking those transcripts whose stability was greater than 1.5 as reported by Vandesompele and collegues^19^.

Newly implemented normalization methods have been integrated in PIPE-T as an updated version of the function *normalizeCtData* of the HTqPCR R package^9^.

### Transcript filtering and imputation

High-throughput data may often contain missing values. For this reason, handling missing values is a crucial step of any RT-qPCR analysis^5,6^. The simplest solution for handling missing values would be to exclude from the analysis any transcript with at least one missing value. In such a case, missing values do not represent a problem anymore because they are removed from the analysis. However, this approach could filter out a considerable number of potential useful transcripts. Another solution would be to take every transcript no matter of the number of missing values. In such a case, all potential useful transcripts are taken into account for subsequent analysis, but the probability of making an error increases with the number of missing values^6^. In the literature, there is a wide accepted approach that consists in keeping transcripts with a reasonable number of missing values and filtering out those exceeding this threshold^6^. Transcripts that do not exceed the threshold are imputed using a suitable method. In the literature, several imputation methods have been proposed^20^.

PIPE-T offers a slider that the user can move to specify the maximum percentage of missing values admissible for a specific transcript. PIPE-T allows filtering transcripts using a user-defined percentage of missing values and/or a user-defined list of transcripts to be removed by using the *filterCtData* function of the HTqPCR package^9^.

In addition, PIPE-T gives the possibility of selecting one of three well-known imputation methods. These methods are:

- KNN
- Mestdagh
- Cubic

KNN and Cubic imputation methods were already implemented in the *impute* and *zoo* R packages.

Mestdagh is an imputation method that substitutes a missing Ct value with a numeric value obtained adding one cycle to the highest Ct value across samples^7^. This method has already been described in other reports^5^. This method assumes that missing values depends on the low or null abundance of the transcript in the sample.

### Differential expression analysis

Differential expression is a very popular analysis for identifying candidate transcripts whose expression can discriminate between two predefined conditions. Among the methods eligible for a differential expression analysis^21^, PIPE-T offers the possibility of choosing between three approaches:

- T-test^21^.
- Two sample Wilcoxon test^21^.
- Rank Product^22^.

T-test and two sample Wilcoxon test are among the most used statistical tests to perform a differential expression analysis^21^. Tests are implemented by *ttestCtData* and *mannwhitneyCtData* functions of the HTqPCR R package^9^. For the t-test and the two sample Wilcoxon test, PIPE-T offers the possibility of setting up six distinct parameters, which include: the types of alternative hypothesis to assess significance, the choice of a paired or an unpaired analysis, the presence in the data of replicated transcripts, the choice of a more or less stringent analysis, and the choice of the method for adjusting p-values in case of multiple hypothesis testing.

Rank Product is a popular method originating from a biological reasoning^22^. Rank Product is carried out using *RP* function of RankProd R package^23^.

If users do not specify any differential expression analysis method, PIPE-T allows them to select an option named NONE. In this case, no differential expression analysis is performed on the data.

### Data visualization and outputting

Quality assessment of RT-qPCR data is crucial for enhancing the accuracy of the results and the reliability of the conclusions^2^. HTqPCR provides several visualization options for assessing the quality of qPCR data, which include histograms, boxplots, density distributions, and scatter plots^9^. PIPE-T uses two boxplot visualizations showing the distribution of the expression values across all samples. The boxplots show the distribution of expression values before and after data normalization, respectively. The visual inspection of the two boxplots is used as qualitative assessment of the normalization procedure because boxplots show the noise reduction comparing the data before and after data normalization^8^. Empirical Cumulative Distribution Function (ECDF) is also used in the literature for measuring noise reduction as an effect of data normalization^8,10^. PIPE-T computes and plots ECDF before and after data normalization by using *ecdf* function of the stats R package^14^. The significance of the difference between the two ECDF curves is estimated by Kolmogorov-Smirnov test and p-value is reported on top of the figure and in the standard output.

Tabular output files include raw data, filtered data, imputed data and statistics to assess differential expression. A detailed description of the row and column names can be found in HTqPCR and RankProd R packages documentation. A detailed description of visualization, sharing, and workflow integration using Galaxy graphical interface can be found in the Galaxy documentation.

## Results

We tested the ability of PIPE-T of analyzing RT-qPCR data using two example datasets whose tab-separated text files were available in the Gene Expression Omnibus (GEO) with accession identifiers GSE25552 and GSE43000. Datasets were relative to two published studies on various metastatic tumors^24^ and non-small cell lung (NSCL) cancers^25^. The first study reported the results of the analysis of sixteen different tumors including Lung, Renal, Colon, Sarcoma, Ovarian, and Head and neck squamous cell carcinoma^24^. The second study reported the results of the analysis of forty-four NSCL tumor samples^25^. We carried out PIPE-T analysis of both datasets on a test Galaxy instance version 19.01, installed in a local Linux machine. Parameter settings for the two analyses have been taken from the original publications when available. When the parameters were not specified we selected them arbitrarily.

### Various metastatic cancers

**Figure 2:**
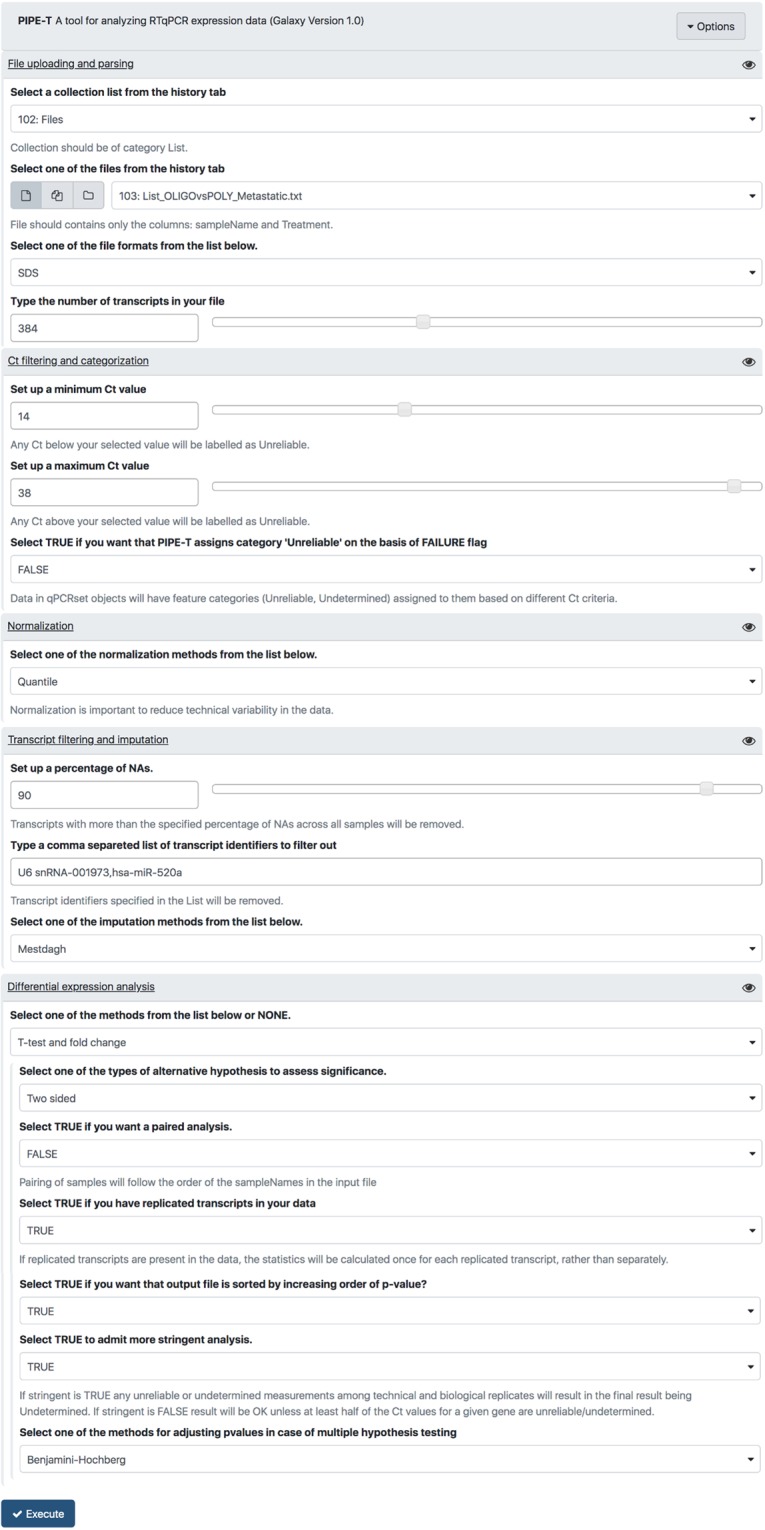
Parameter settings used for the analysis of metastatic cancer data. PIPE-T wrapper interface with the settings used for the analysis of the metastatic cancer dataset. Input files have already been uploaded using the “Upload File from your computer” tool.

We downloaded input tab-delimited files from GEO and we added a SDS version 2.4 format header to each of these files because it lacked. Input files contained experimental data for 384 microRNAs. We coupled RT-qPCR data with information about tumor status, which was oligometastatic (OLIGO) for ten out of sixteen patients and polymetastatic (POLY) for the remaining six patients. File names and tumor status were organized into a tab-delimited text file. The newly created file and the sixteen tab-separated text files were uploaded in Galaxy as fileTreatment and ListOfFile through “Upload File from your computer” tool. Analysis was carried out with parameters settings reported in Fig. 2.

**Figure 3:**
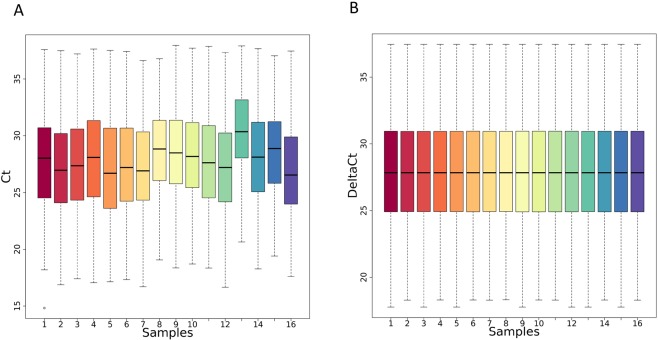
Qualitative assessment of the noise reduction for metastatic cancer data. Box plots show the distribution of Ct values in metastatic cancer samples after Ct filtering and categorization (Panel A) and after normalization (Panel B) procedures. Each box plot is relative to a sample.

**Figure 4:**
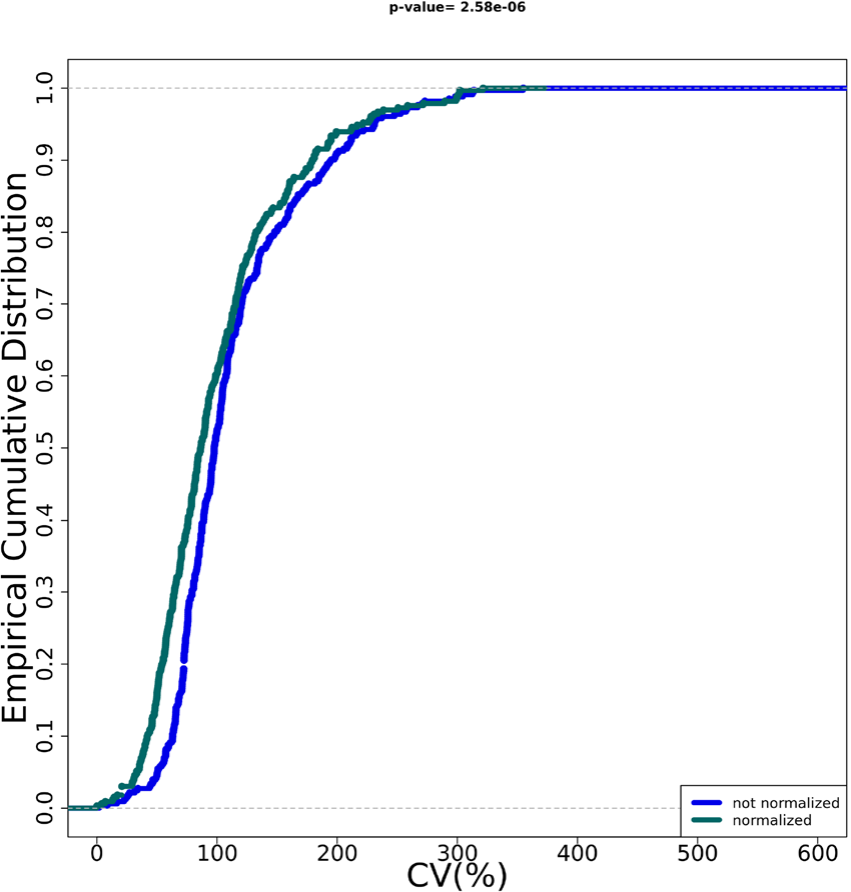
Quantitative assessment of the noise reduction for metastatic cancer data. ECDFs (y axis) and coefficient of variation (CV) is displayed for the metastatic cancer samples after Ct filtering and categorization (blue line) and after normalization (green line) procedures. Kolmogorov-Smirnov test assessing the significance of the separation between the curves and p value is reported on top of the plot.

Our tool successfully completed the execution, returning seven output files (see Tables S1–S4 and Figs S1–S3). Boxplots and EDCF before and after data normalization as well as the significant genes and statistics reported by the differential expression analysis procedure are depicted in Figs 3, 4, and Table 1, respectively.

**Table 1:** Significant genes estimated by the differential expression analysis procedure in metastatic cancer dataset.

genes^a^	t.test^b^	p.value^c^	adj.p.value^d^	ddCt^e^	FC^f^	meanCalibrator^g^	meanTarget^h^	categoryCalibrator^i^	categoryTarget^j^
hsa-miR-200c-4395411	3.110	0.011	0.449	−4.414	21.324	25.793	21.378	OK	OK
hsa-miR-375-4373027	2.558	0.026	0.560	−3.943	15.381	27.514	23.570	OK	OK
hsa-miR-141-4373137	2.288	0.043	0.563	−3.640	12.467	28.392	24.751	OK	OK
hsa-miR-654-3p-4395350	2.810	0.019	0.488	−3.008	8.045	34.747	31.739	Undetermined	Undetermined
hsa-miR-135b-4395372	2.937	0.013	0.449	−2.916	7.546	28.848	25.932	OK	OK
hsa-miR-200b-4395362	2.912	0.014	0.449	−2.516	5.722	24.808	22.291	OK	OK
hsa-miR-410-4378093	2.299	0.047	0.563	−2.285	4.873	31.701	29.417	OK	OK
hsa-miR-323-3p-4395338	2.209	0.049	0.563	−1.988	3.966	30.228	28.240	OK	OK
hsa-miR-370-4395386	2.604	0.021	0.488	−1.686	3.218	27.286	25.600	OK	OK
hsa-miR-642-4380995	2.807	0.015	0.449	−1.673	3.188	31.283	29.610	OK	OK
hsa-miR-127-3p-4373147	2.338	0.035	0.563	−1.512	2.853	26.167	24.655	OK	OK
hsa-miR-212-4373087	4.008	0.001	0.449	−1.422	2.680	27.481	26.059	OK	OK
hsa-miR-628-5p-4395544	−2.530	0.032	0.563	1.097	0.467	29.281	30.378	OK	Undetermined
hsa-miR-125a-3p-4395310	−2.790	0.017	0.474	1.191	0.438	29.835	31.026	OK	OK
hsa-miR-328-4373049	−2.592	0.028	0.563	1.215	0.431	27.772	28.987	OK	OK
hsa-miR-886-3p-4395305	−2.311	0.042	0.563	1.225	0.428	24.007	25.232	OK	OK
hsa-miR-140-5p-4373374	−2.880	0.012	0.449	1.245	0.422	23.457	24.702	OK	OK
hsa-miR-29c-4395171	−2.926	0.015	0.449	1.339	0.395	23.351	24.691	OK	OK
hsa-miR-140-3p-4395345	−3.118	0.008	0.449	1.410	0.376	26.526	27.935	OK	OK
hsa-miR-570-4395458	−2.305	0.038	0.563	1.460	0.363	34.499	35.959	Undetermined	Undetermined
hsa-miR-489-4395469	−3.029	0.009	0.449	1.522	0.348	26.810	28.332	OK	OK
hsa-miR-545-4395378	−3.097	0.008	0.449	2.067	0.239	31.847	33.914	Undetermined	Undetermined
hsa-miR-502-5p-4373227	−3.107	0.009	0.449	3.300	0.102	29.814	33.113	Undetermined	Undetermined

^a^Name of the microRNA in the card. Data are calculated by ttestCtData function of the HTqPCR package.

Calibrator is the treatment group of the first sampleName in fileTreatment. Target is the alternative treatment group. In our example, Calibrator is OLIGO and Target is POLY.

^b^Value of t statistics.

^c^Significance of the difference between the mean of expression of the treatment groups. MicroRNAs are ordered by p value.

^d^P value adjusted for multiple hypothesis testing.

^e^Delta delta Ct value.

^f^Fold change value calculated as 2^−ddCt^. FC greater than 2 and lower than 0.5 have been reported.

^g^Average expression of the microRNA in the Calibrator group.

^h^Average expression of the microRNA in the target group.

^i^Category of the Ct values (“OK”, “Undetermined”) across the samples of calibrator group.

^j^Category of the Ct values (“OK”, “Undetermined”) across the samples of target group.

We found 12 significantly upregulated and 11 downregulated microRNAs in polymetastatic tumors (p value < 0.05 and FC > 2 or FC < 0.5; Table 1).

Interestingly, among the significantly modulated microRNAs reported in the Lussier and coworkers manuscript^24^, 11 out of 12 microRNAs were consistently up regulated in polymetastatic tumors and 8 out of 11 microRNAs were consistently upregulated in oligometastatic tumors. Any difference between our findings and those reported by Lussier and collegues^24^ are probably due to the different approaches used in the experiments to filter and handle missing values. Lussier and collegues did not report any information about filtering based on the percentage of missing values or the application of any method for handling missing or unreliable Ct values. These results provide the first evidence that PIPE-T is able to correctly analyze RT-qPCR expression data.

### Non-small cell lung cancer

**Figure 5:**
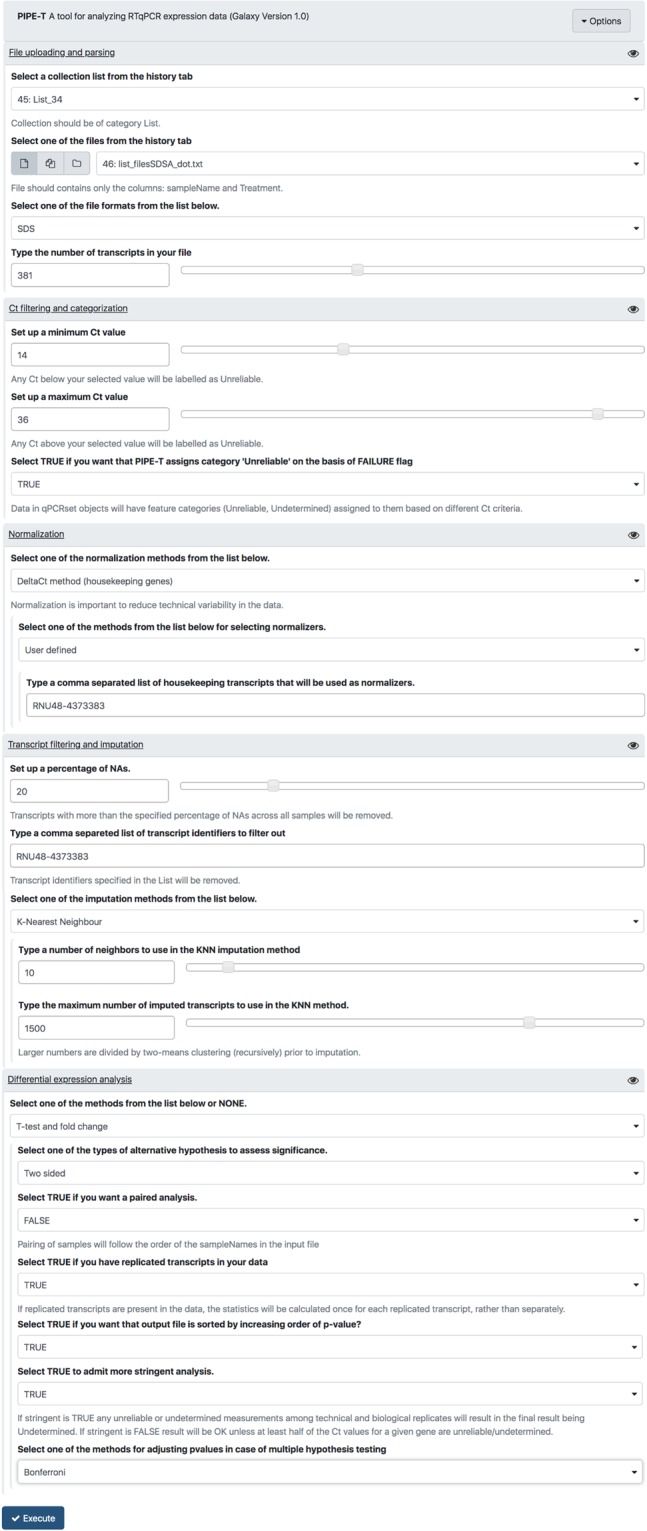
Parameter settings used for the analysis of NSLC data. PIPE-T wrapper interface with the settings used for the analysis of the NSLC dataset. Input files have already been uploaded using the “Upload File from your computer” tool.

NSCL input files were compliant with SDS format version 2.3 and reported experimental data for 381 microRNAs. Since the downloaded files used a comma as decimal separator, each comma was replaced with a dot before running PIPE-T. RT-qPCR data were coupled with histological data provided in the original publication^25^, which refer to twenty lung adenocarcinoma (LA) and twenty-four squamous cell lung cancer (SCLC). File names and tumor subtypes were organized into a text file. We uploaded the newly created file as fileTreatment, and the forty-four tab-separated text files as ListOfFile. Analysis was carried out with the parameter settings reported in Fig. 5.

**Figure 6:**
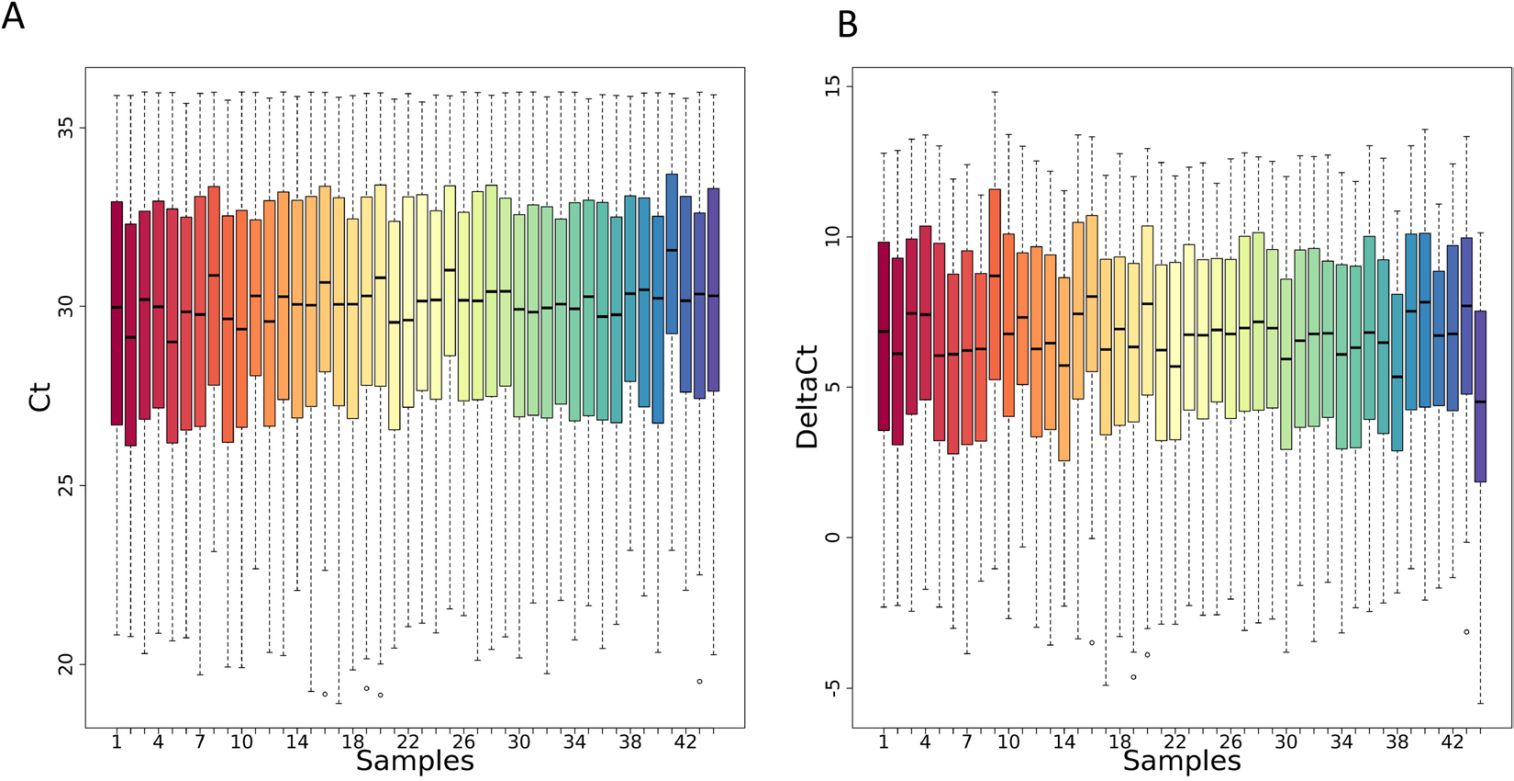
Qualitative assessment of the noise reduction for NSLC data. Box plots show the distribution of Ct values in NSLC samples after Ct filtering and categorization (Panel A) and after normalization (Panel B) procedures. Each box plot is relative to a sample.

**Figure 7:**
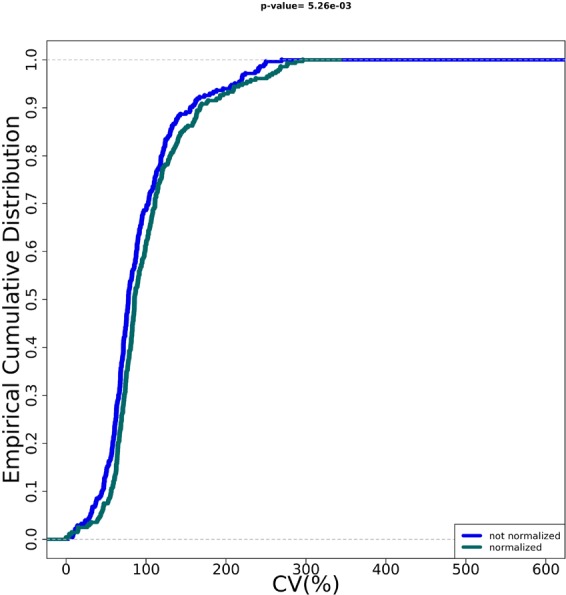
Quantitative assessment of the noise reduction for NSLC data. ECDFs (y axis) and coefficient of variation (CV) is displayed for the NSLC samples after Ct filtering and categorization (blue line) and after normalization (Green line) procedures. Kolmogorov-Smirnov test assessing the significance of the separation between curves and p value is reported on top of the plot.

Our tool successfully completed the execution returning seven output files (see Tables S5–S8 and Figs S4–S6). Boxplots and EDCF before and after normalization, as well as the significant microRNAs identified by the differential expression analysis procedure, are depicted in Figs 6, 7, and Table 2, respectively.

**Table 2:** Significant genes estimated by the differential expression analysis procedure in the NSLC dataset.

genes^a^	t.test^b^	p.value^c^	adj.p.value^d^	ddCt^e^	FC^f^	meanCalibrator^g^	meanTarget^h^	categoryCalibrator^i^	categoryTarget^j^
hsa-miR-205-4373093	5.190	0.000	0.001	−4.411	21.272	8.013	3.602	Undetermined	Undetermined
hsa-miR-375-4373027	−4.079	0.000	0.038	2.065	0.239	3.354	5.418	OK	OK
hsa-miR-422a-4395408	3.854	0.000	0.069	−1.418	2.673	8.215	6.796	OK	OK
hsa-miR-149-4395366	3.758	0.001	0.094	−2.322	5.000	7.393	5.071	OK	Undetermined
hsa-miR-708-4395452	3.634	0.001	0.135	−2.044	4.123	5.995	3.952	OK	OK
hsa-miR-204-4373094	3.440	0.001	0.232	−1.587	3.004	9.177	7.590	Undetermined	Undetermined
hsa-miR-483-5p-4395449	3.376	0.002	0.285	−1.397	2.634	10.019	8.622	Undetermined	Undetermined
hsa-miR-127-3p-4373147	2.984	0.005	0.918	−1.166	2.245	6.256	5.090	OK	OK
hsa-miR-196b-4395326	2.915	0.006	1.000	−1.933	3.818	9.416	7.483	Undetermined	Undetermined
hsa-miR-202-4395474	2.922	0.006	1.000	−1.061	2.087	10.305	9.244	Undetermined	Undetermined
hsa-miR-494-4395476	2.669	0.011	1.000	−1.165	2.242	3.657	2.492	OK	OK
hsa-miR-376a-4373026	2.628	0.012	1.000	−1.093	2.133	10.615	9.522	Undetermined	Undetermined
hsa-miR-376c-4395233	2.624	0.013	1.000	−1.299	2.460	9.324	8.025	OK	Undetermined
hsa-miR-130b-4373144	2.575	0.014	1.000	−1.071	2.100	8.247	7.176	Undetermined	Undetermined
hsa-miR-203-4373095	2.131	0.039	1.000	−1.098	2.140	4.716	3.618	Undetermined	OK
hsa-miR-194-4373106	−2.089	0.046	1.000	1.182	0.441	8.508	9.690	OK	Undetermined

^a^Name of the microRNA in the card. Data are calculated by ttestCtData function of the HTqPCR package.

Calibrator is the treatment group of the first sampleName in fileTreatment. Target is the alternative treatment group. In our example, Calibrator is LA and Target is SCLC.

^b^Value of t statistics.

^c^Significance of the difference between the mean of expression of the treatment groups. MicroRNAs are ordered by p value.

^d^P value adjusted for multiple hypothesis testing.

^e^Delta delta Ct value.

^f^Fold change value calculated as 2^−ddCt^. FC greater than 2 and lower than 0.5 are reported.

^g^Average expression of the microRNA in the Calibrator group.

^h^Average expression of the microRNA in the target group.

^i^Category of the Ct values (“OK”, “Undetermined”) across the samples of calibrator group.

^j^Category of the Ct values (“OK”, “Undetermined”) across the samples of target group.

We found 16 significantly modulated microRNAs (p value < 0.05 and FC > 2 or FC < 0.5; Table 2). Interestingly, miR-205, miR-149, miR-422a, and miR-708 were significantly upregulated in SCLC and miR-375 was significantly upregulated in LA in accordance with the results of the original manuscript^25^. Any difference of fold change or p-value between our study and that by Molina-Pinelo and collegues^25^ can be explained by the different handling of missing values. Authors did not report their approach to missing or unreliable Ct values. In spite of three small differences, our results provide evidences that PIPE-T is able to correctly analyze RT-qPCR expression data.

## Conclusions

We developed PIPE-T, a new Galaxy tool that offers several state-of-the-art options for parsing, filtering, normalizing, imputing, and analyzing RT-qPCR expression data. Integration of PIPE-T into Galaxy allows researchers with strong bioinformatic background, as well as those without any programming expertise, to perform complex analysis in a simple to use, transparent, accessible, reproducible, and user-friendly environment.

## Availability of Supporting Source Code and Requirements

Project name: Pipe-t

Project home page: https://github.com/igg-molecular-biology-lab/pipe-t (2019)^26^

Operating system(s): Linux (Galaxy), and platform independent

Programming language: R

Other requirements: Galaxy

License: GNU GPL

PIPE-T is available on the Main Tool Shed^15^ at the link^27^, on the Docker^28^ at the link^29^ and on the web^30^ at the link^31^. PIPE-T code is freely available on GitHub at the link https://github.com/igg-molecular-biology-lab/pipe-t (2019)^26^.

PIPE-T has the following dependencies:

<requirements>

<requirement type = “package” version = “3.5.0”>r-base</requirement>

<requirement type = “package” version = “7.2.0”>libgcc</requirement>

<requirement type = “package” version = “1.36.0”>bioconductor-htqpcr</requirement>

<requirement type = “package” version = “3.8.0”>bioconductor-rankprod</requirement>

<requirement type = “package” version = “1.56.0”>bioconductor-impute</requirement>

<requirement type = “package” version = “1.11.0”>r-bbmisc</requirement>

<requirement type = “package” version = “1.8.4”>r-psych</requirement>

<requirement type = “package” version = “1.8_3”>r-zoo</requirement>

</requirements>

If Conda^32^ is installed and enabled, Galaxy locates and resolves any tool dependencies automatically during tool installation.

## Data availability

The tab-separated text files included in the ListOfFile collections of the two example applications are available in GEO repository with accession numbers: GSE25552 and GSE43000. A detailed documentation, step-by-step tool installation instructions, configuration, example applications are available on GitHub at the link https://github.com/igg-molecular-biology-lab/pipe-t (2019)^26^.

## Supplementary information

Supplementary information

Supplementary Dataset

## References

[1] Pabinger S, Rodiger S, Kriegner A, Vierlinger K, Weinhausel A (2014). A survey of tools for the analysis of quantitative PCR (qPCR) data. Biomol Detect Quantif.

[2] Derveaux S, Vandesompele J, Hellemans J (2010). How to do successful gene expression analysis using real-time PCR. Methods.

[3] VanGuilder HD, Vrana KE, Freeman WM (2008). Twenty-five years of quantitative PCR for gene expression analysis. Biotechniques.

[4] McCall MN, McMurray HR, Land H, Almudevar A (2014). On non-detects in qPCR data. Bioinformatics.

[5] de Ronde MWJ, Ruijter JM, Moerland PD, Creemers EE, Pinto-Sietsma SJ (2018). Study Design and qPCR Data Analysis Guidelines for Reliable Circulating miRNA Biomarker Experiments: A Review. Clin Chem.

[6] Marabita F (2016). Normalization of circulating microRNA expression data obtained by quantitative real-time RT-PCR. Brief Bioinform.

[7] Mestdagh P (2014). Evaluation of quantitative miRNA expression platforms in the microRNA quality control (miRQC) study. Nat Methods.

[8] Mestdagh P (2009). A novel and universal method for microRNA RT-qPCR data normalization. Genome Biol.

[9] Dvinge H, Bertone P (2009). HTqPCR: high-throughput analysis and visualization of quantitative real-time PCR data in R. Bioinformatics.

[10] D’haene B, Mestdagh P, Hellemans J, Vandesompele J (2012). miRNA expression profiling: from reference genes to global mean normalization. Methods Mol Biol.

[11] Taylor SC (2019). The Ultimate qPCR Experiment: Producing Publication Quality, Reproducible Data the First Time. Trends Biotechnol.

[12] Goecks J, Nekrutenko A, Taylor J (2010). Galaxy: a comprehensive approach for supporting accessible, reproducible, and transparent computational research in the life sciences. Genome Biol.

[13] Blankenberg D (2010). Galaxy: a web-based genome analysis tool for experimentalists. Curr Protoc Mol Biol.

[14] R Core Team. R: A language and environment for statistical computing; Vienna, https://www.R-project.org (2019).

[15] Blankenberg D (2014). Dissemination of scientific software with Galaxy Tool Shed. Genome Biol.

[16] Meyer SU, Pfaffl MW, Ulbrich SE (2010). Normalization strategies for microRNA profiling experiments: a ‘normal’ way to a hidden layer of complexity?. Biotechnol Lett.

[17] Zeileis A, Grothendieck G (2005). Zoo: S3 Infrastructure for Regular and Irregular Time Series. In. Journal of Statistical Software.

[18] Perkins JR (2012). ReadqPCR and NormqPCR: R packages for the reading, quality checking and normalisation of RT-qPCR quantification cycle (Cq) data. BMC Genomics.

[19] Vandesompele Jo, De Preter Katleen, Pattyn Filip, Poppe Bruce, Van Roy Nadine, De Paepe Anne, Speleman Frank (2002). Genome Biology.

[20] Yadav ML, Roychoudhury B (2018). Handling missing values: A study of popular imputation packages in R. In. Knowledge-Based Systems.

[21] Andrew H, Florence G, Kibria GB (2015). Methods for identifying differentially expressed genes: An empirical comparison. Journal of Biometrics & Biostatistics.

[22] Breitling R, Armengaud P, Amtmann A, Herzyk P (2004). Rank products: a simple, yet powerful, new method to detect differentially regulated genes in replicated microarray experiments. FEBS Lett.

[23] Hong F (2006). RankProd: a bioconductor package for detecting differentially expressed genes in meta-analysis. Bioinformatics.

[24] Lussier YA (2011). MicroRNA expression characterizes oligometastasis(es). PLoS One.

[25] Molina-Pinelo S (2014). MicroRNA-dependent regulation of transcription in non-small cell lung cancer. PLoS One.

[26] GitHub, https://github.com/igg-molecular-biology-lab/pipe-t Accessed 20 May (2019).

[27] Galaxy main tool shed repository, https://davidecangelosi@toolshed.g2.bx.psu.edu/repos/davidecangelosi/pipe_t Accessed 20 May (2019).

[28] Merkel D (2014). Docker: lightweight linux containers for consistent development and deployment. Linux Journal.

[29] Docker, https://hub.docker.com/r/davidecangelosi/galaxy-pipe-t Accessed 20 May (2019).

[30] Tangaro, M. A. *et al*. Laniakea: an open solution to provide “Galaxy on-demand” instances over heterogeneous cloud infrastructures. *bioRxiv*, 472464, 10.1101/472464 (2018). 10.1093/gigascience/giaa033 PMC7136032 32252069

[31] Live Galaxy Instance website, http://igg.cloud.ba.infn.it/galaxy Accessed 22 October (2019).

[32] Gruning B (2018). Bioconda: sustainable and comprehensive software distribution for the life sciences. Nat Methods.

