# Supplementary material for: PIPE-T: a new Galaxy tool for the analysis of RT-qPCR expression data
Source: Sci Rep. 2019 Nov 26;9:17550. doi: 10.1038/s41598-019-53155-9 (PMC6879478; doi:10.1038/s41598-019-53155-9)
Supplement: Supplementary file 1 — Supplementary information [file 41598_2019_53155_MOESM1_ESM.pdf]

PIPE-T: a new Galaxy tool for the analysis of RT-qPCR expression data

Nicolò Zanardi, Martina Morini, Marco Antonio Tangaro, Federico Zambelli, Maria Carla Bosco, Luigi Varesio, and Alessandra Eva, Davide Cangelosi

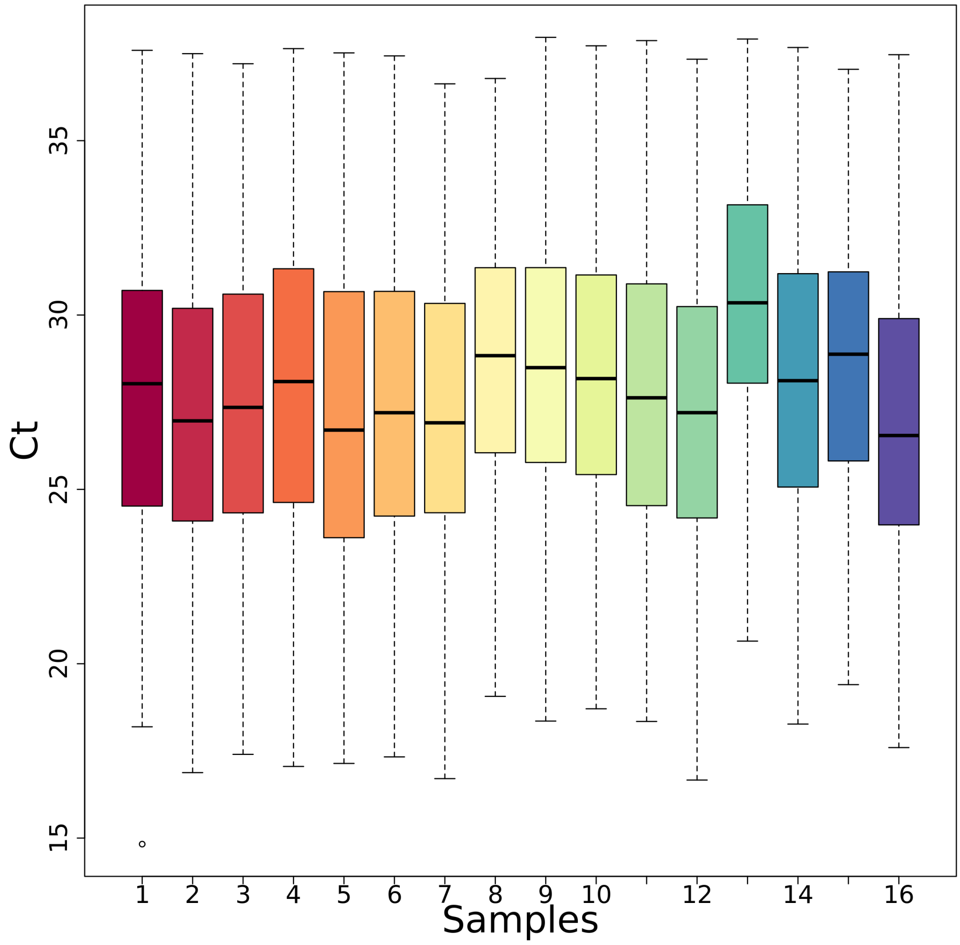

Figure S1. Distribution of the Ct values for metastatic cancer dataset

PIPE-T output PNG file showing the distribution of Ct values after Ct filtering and categorization procedure. Each box plot is relative to a sample.

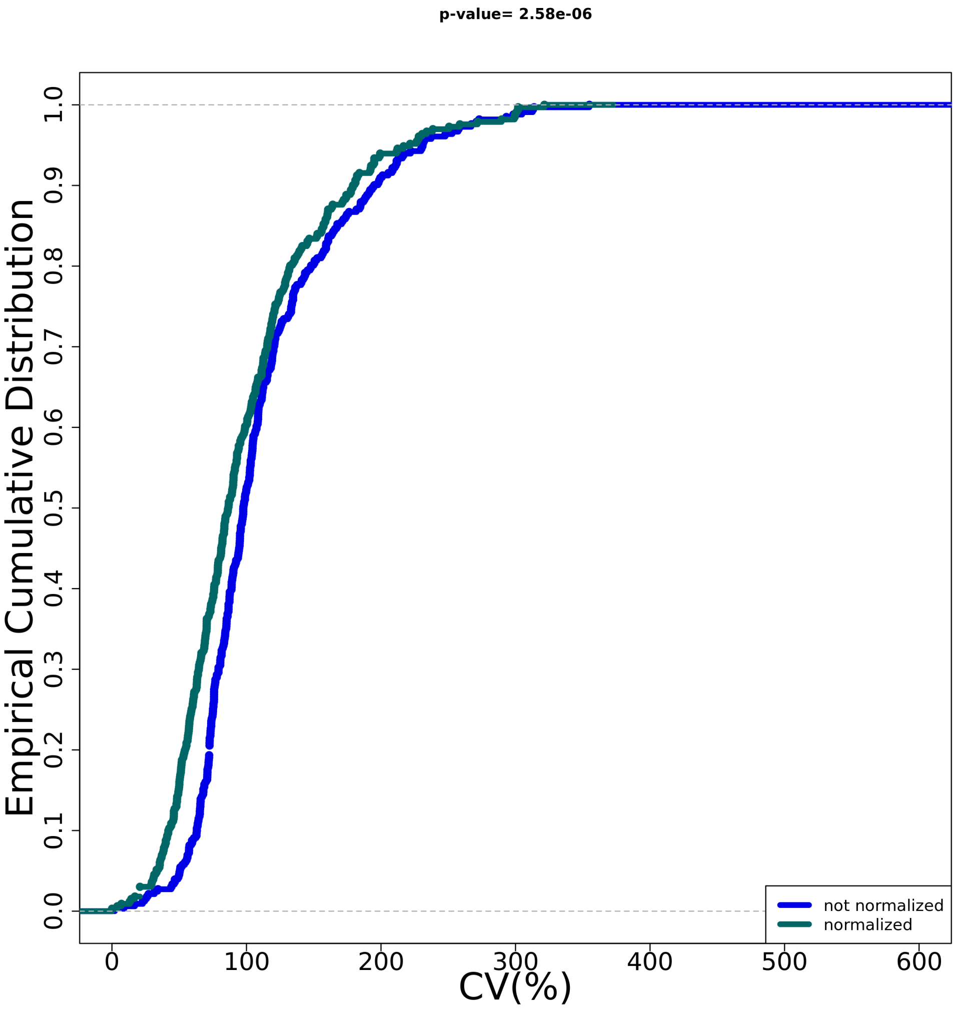

Figure S2. Quantitative assessment of the noise reduction for metastatic cancer dataset

PIPE-T output PNG file showing ECDFs (y axis) and coefficient of variation (CV) after Ct filtering and categorization (blue line) and after normalization (Green line) procedures. Kolmogorov-Smirnov test assessing the significance of the separation between curves and p value is reported on top of the plot.

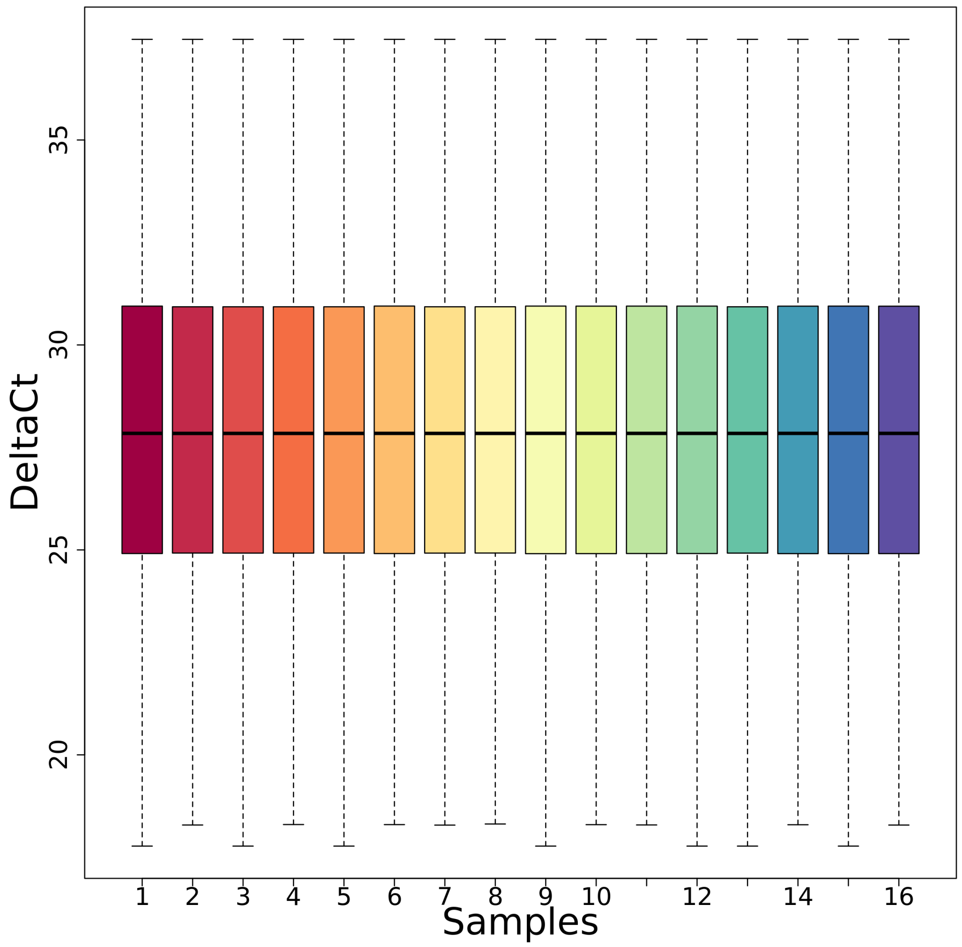

Figure S3. Distribution of the normalized expression values for metastatic cancer dataset

PIPE-T output PNG file showing the distribution of Ct values in after normalization procedure. Each box plot is relative to a sample.

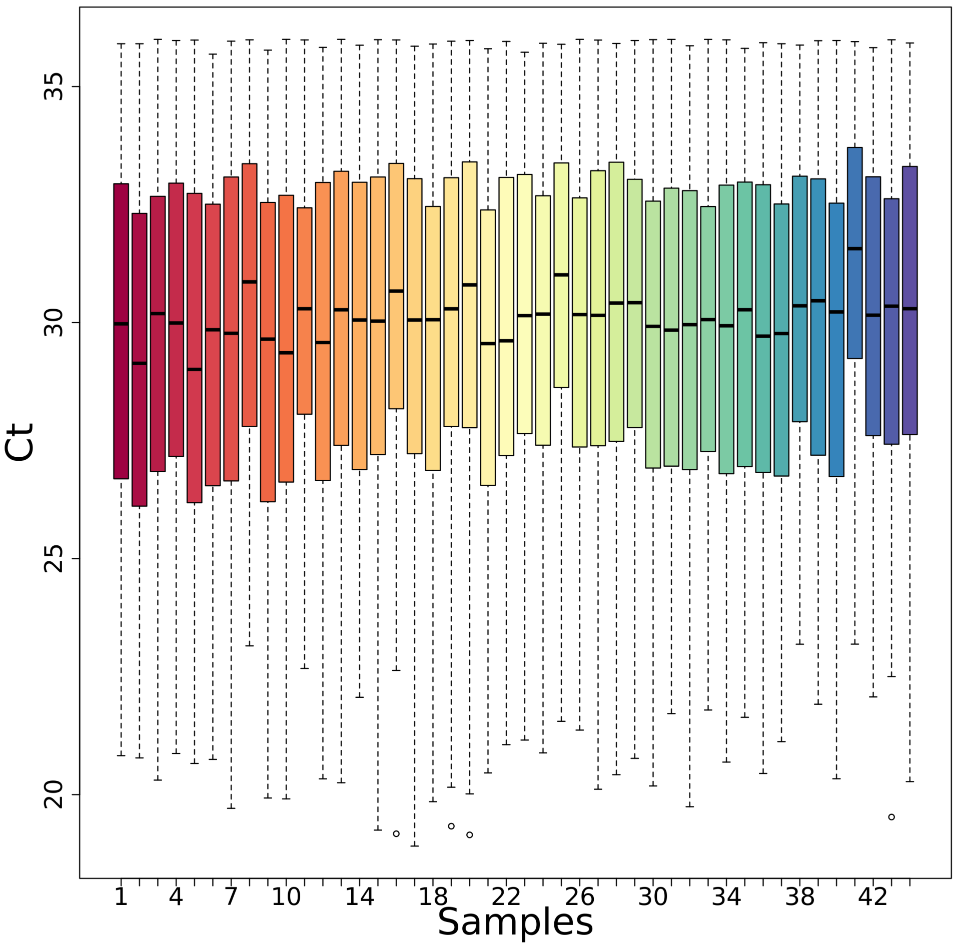

Figure S4. Distribution of the Ct values for NSLC dataset

PIPE-T output PNG file showing the distribution of Ct values after Ct filtering and categorization procedure. Each box plot is relative to a sample.

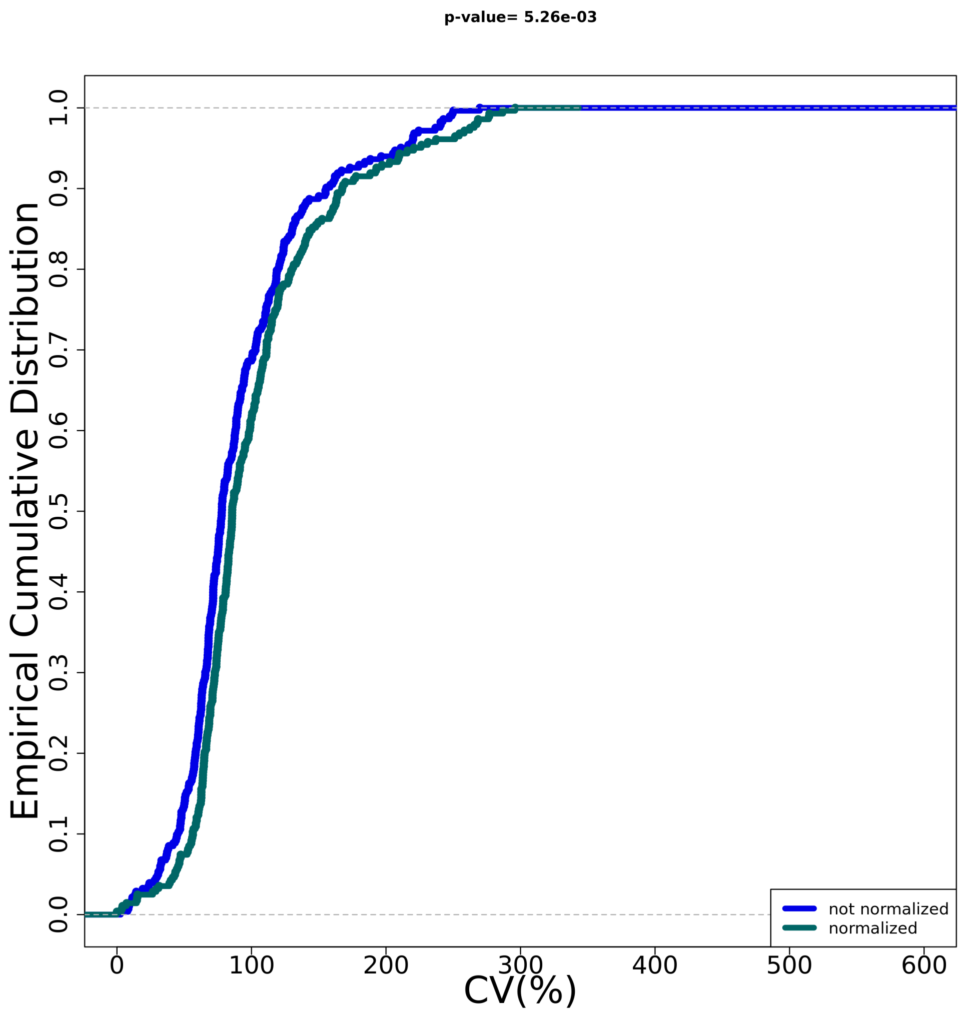

Figure S5. Quantitative assessment of the noise reduction for NSLC dataset

PIPE-T output PNG file showing ECDFs (y axis) and coefficient of variation (CV) after Ct filtering and categorization (blue line) and after normalization (Green line) procedures. Kolmogorov-Smirnov test assessing the significance of the separation between curves and p value is reported on top of the plot.

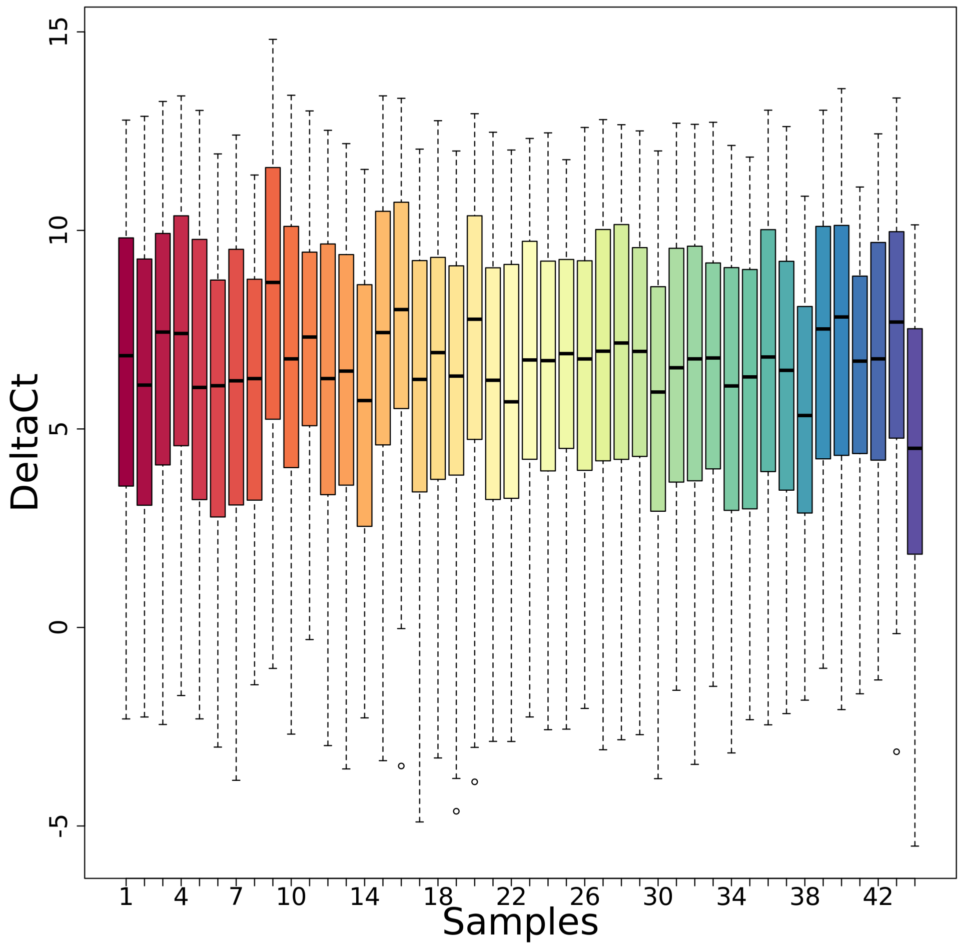

Figure S6. Distribution of the normalized expression values for NSLC dataset

PIPE-T output PNG file showing the distribution of Ct values in after normalization procedure. Each box plot is relative to a sample.
